# How Long Are Cancer Patients Waiting for Oncological Therapy in Poland?

**DOI:** 10.3390/ijerph15040577

**Published:** 2018-03-23

**Authors:** Karolina Osowiecka, Monika Rucinska, Jacek J. Nowakowski, Sergiusz Nawrocki

**Affiliations:** 1 Department of Radiation Oncology, Hospital of the Ministry of Internal Affairs with Warmia and Mazury Oncology Center in Olsztyn, Al. Wojska Polskiego 37, 10-228 Olsztyn, Poland; m_rucinska@poczta.onet.pl; 2 Department of Public Health, Epidemiology and Microbiology, University of Warmia and Mazury in Olsztyn, Al. Warszawska 30, 10-082 Olsztyn, Poland; 3 Department of Oncology, University of Warmia and Mazury in Olsztyn, Al. Wojska Polskiego 37, 10-228 Olsztyn, Poland; 4 Department of Ecology and Environmental Protection, University of Warmia and Mazury in Olsztyn, Pl. Łódzki 3, 10-727 Olsztyn, Poland; jacek.nowakowski@uwm.edu.pl; 5 Department of Oncology and Radiotherapy, Medical University of Silesia in Katowice, Ul. Ceglana 35, 40-514 Katowice, Poland; sergiusz@cyberia.pl

**Keywords:** cancer, waiting time, diagnosis, health services

## Abstract

Background: The five-year relative survival rate in Poland is approximately 10% lower compared with the average for Europe. One of the factors that may contribute to the inferior treatment results in Poland could be the long time between cancer suspicion and the beginning of treatment. The aim of the study was to determine the real waiting time for cancer diagnosis and treatment in Poland. Methods: The study was carried out in six cancer centers on a group of 1373 patients, using a questionnaire to interview patients. The median waiting time was estimated as follows: (A) from suspicion (the date of the first visit, with symptoms, to a doctor or a preventive or screening test) until histopathological diagnosis; (B) from suspicion until initial treatment; and (C) from diagnosis until initial treatment. Results: The median times from suspicion to treatment, from suspicion to diagnosis, and from diagnosis to treatment, were 10.6, 5.6, and 5.0 weeks, respectively. Using multivariate analysis, the strongest influence was estimated, in a case of tumor localization, to be the method of initial treatment and facilities. Conclusion: The waiting time for cancer treatment in Poland is too long. The highest influence on waiting time was determined, in the case of tumors, as the type of cancer and factors related to the health care system.

## 1. Introduction

Cancer is a significant global health problem. In Poland, cancer is currently the second leading cause of death—after circulatory system diseases—and is likely to become the leading cause of death within 10 years [1,2,3]. Currently, cancer is the dominant cause of premature deaths among Polish women between the ages of 30 to 69 years [3]. According to the most accurate EUROCARE 5 (European Cancer Registry 5) analysis, the five-year relative survival rate in Poland is approximately 10% lower when compared with the average for Europe (42.7% vs. 54.6%, respectively) [4]. The inferior cancer treatment results in Poland could be attributed to late cancer detection at an advanced clinical stage. Another reason for this could be that the resources available in Poland for medical expenses are probably too low. In Poland, the expenditure on oncological therapy per capita is approximately one third of the average expenditure of the European Union (EU) (37 vs. 102 euro per year, respectively) [2]. Therefore, this could prolong the time from the cancer suspicion to the beginning of treatment. The Organisation for Economic Co-operation and Development [5] showed that the long waiting time for diagnosis and treatment has the most influence on the limited availability of effective health care in Poland. Poland holds the penultimate place in mortality rates among EU countries. In 2012, the mortality/incidence ratio in Poland was 0.60 and—together with Romania and Greece—was the highest in the EU. In countries where the expenditure on oncological treatment was the highest, the mortality/incidence ratio was approximately 0.40 [2].

In many countries, such as Great Britain, Scandinavia, and the Czech Republic, the waiting time for treatment is regulated by law. In Great Britain, the time between the general practitioner’s and specialist’s visits cannot exceed 14 days, and the time from diagnosis to treatment cannot exceed 31 days. The maximum time from the visit to a general practitioner, to the beginning of treatment is 62 days. The established waiting time for treatment in Great Britain (nine weeks) was used in this study, because of the similarity of the organization and financing of the Polish public healthcare system to that of the British healthcare system [6]. To our knowledge, the maximum waiting time for oncological therapy in Poland has not been determined. This analysis has estimated an accurate waiting time for diagnosis and treatment of cancer in Poland.

We have assumed that the waiting time for the treatment of cancer patients in Poland is too long, and that the median time from the date of the first visit to a doctor to the treatment exceeds nine weeks. The aim of the study was to determine the amount of time that is necessary for a patient to be diagnosed and treated for cancer, and to estimate the influence of different factors on the delay, taking into consideration private medical services (paid for by the patients and not reimbursed).

## 2. Materials and Methods

The study was carried out on a group of 1373 patients who were being treated for malignant neoplasm—from 22 May 2014 to 19 February 2015—in six oncological centers in Poland. There were 11 cancer centers in Poland that were invited to participate—varying in geographical location, size, organization, and financing services. The consent to conduct the study was received from six facilities. Three of these facilities were public, regional provincial oncological centers where treatment was reimbursed with public resources (IA-C), and three of these facilities were private centers: in one, treatment was reimbursed with public resources (IIA) and in two, treatment expenses were not reimbursed but covered by the owners and not by the patients (IIIA-B). The study was sponsored by Fundacja Onkologia 2025.

A questionnaire was prepared specifically for this study and each patient was interviewed individually. The questionnaire was created based on general principles and was validated on a group of 50 patients. The validation procedure included a questionnaire that was carried out twice in a two-week interval, on the same group of patients. The measure of compliance was calculated using Cohen’s Kappa coefficient. The data obtained was supplemented with medical records and hospital databases.

The study protocol was approved by the Local Ethics Committee of the University of Warmia and Mazury, in Olsztyn, Poland. All of the participants had submitted a signed consent form.

**Figure 1:**
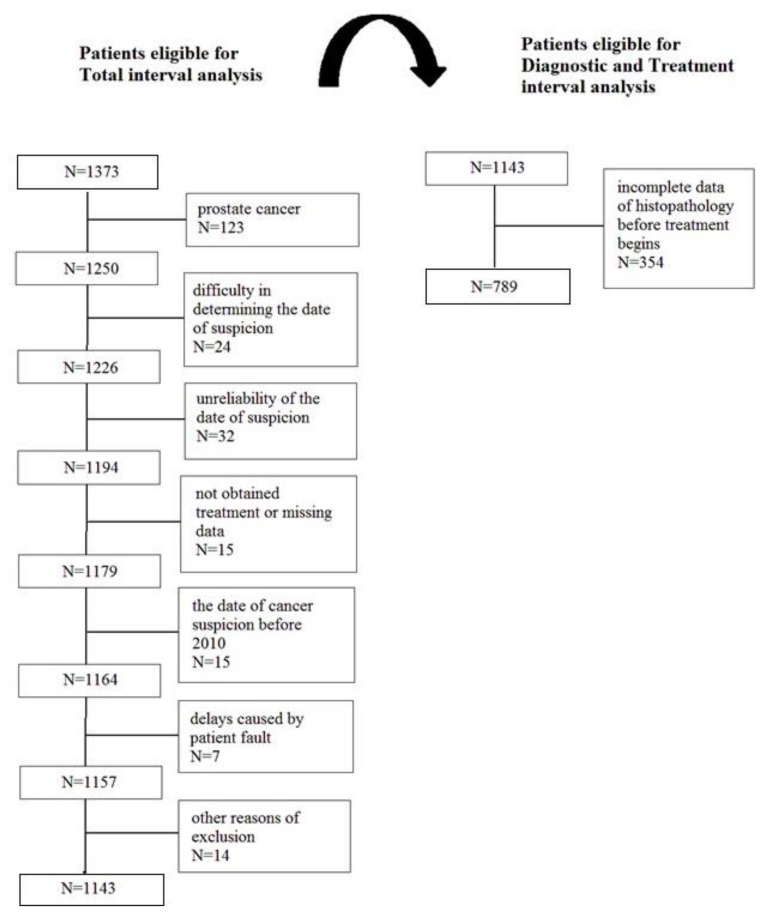
Cohort members: Flow diagram of the selection process.

The patients with prostate cancer diagnoses were excluded from the analysis, as there was no reason to begin the treatment earlier because of the less dynamic development of the cancer in comparison with other types of cancer. There were 230 patients that were excluded from the analysis of the waiting time between the suspicion and the treatment. In addition, 354 patients with incomplete data of histopathology (before the treatment began) were excluded from the analysis of the waiting time between the suspicion and the diagnosis, and between the diagnosis and start of the treatment. In these 354 cases, there was either no histopathological cancer confirmation before the start of the cancer treatment (i.e., an operation without an initial biopsy), or there was no possibility of finding the original histopathological report from the biopsy, or the date of the histopathological confirmation of cancer was unavailable (Figure 1).

**Figure 2:**
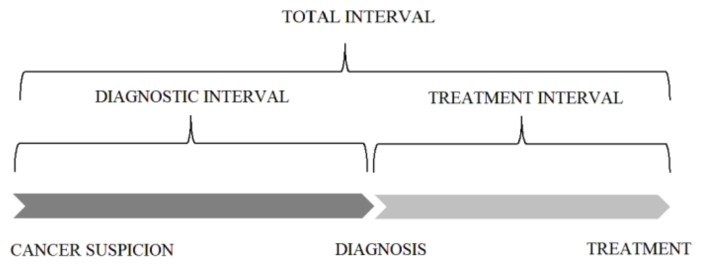
Categorization of intervals in the “patient route”.

There were three periods of waiting times that were measured, namely, from the suspicion to the diagnosis (the diagnostic interval), from the suspicion to the treatment (the total interval), and from the diagnosis to the treatment (the treatment interval) (Figure 2). Cancer suspicion was defined as the date of the beginning of a “patient route”, that is, the date of the first visit, with symptoms, to a doctor; the date of a preventive or screening test in the case of patients without symptoms; or the date of the control visit when the local recurrence of a previously treated cancer or distant metastasis was observed. The diagnosis was defined as the end of the histopathological or cytological examination. The duration of the histopathological examination (from the date of the biopsy to the date of the result from the histopathological analysis) was also evaluated. The treatment was defined as the date of the initial cancer treatment—surgery, radiotherapy or radiochemotherapy, chemotherapy, or hormonal therapy.

### Statistical Analysis

The median, first quartile (Q1), and the third quartile (Q3) of the waiting time distributions were estimated. The validation of the questionnaire was carried out using Cohen’s Kappa. The waiting time distributions were compared with the theoretical normal distribution using the Shapiro–Wilk test. The differences in the waiting time between the subgroups were analyzed with either the Mann–Whitney or the Kruskal–Wallis test, and the Dunn’s test post hoc. The correlation between the waiting time and patient’s age was analyzed using the Spearman correlation coefficient. The generalized linear model (GLM), with the Akaike information criterion (AIC) for gamma distribution and logarithmic function, was used to determine the relationship between the waiting time and a set of predictor variables. The independence of the categorical data was tested using the chi-square test. The logistic regression model was used to determine the relationships between the use of private medical services and other factors—namely, age and education. A *p* value of <0.05 was considered to be significant. The analysis was conducted using STATISTICA software (version 12.5) (StatSoft, Kraków, Poland) and SPSS Statistics 23.0 (IBM, Armonk, NY, USA).

## 3. Results

A total of 1143 patients met the criteria for the analysis of the total interval (from the cancer suspicion to the start of the treatment). The stepwise analyses—of the diagnostic interval (from the cancer suspicion to the histopathological cancer confirmation) and the treatment interval (from the histopathological cancer confirmation to the start of treatment)—were carried out on 789 patients (Figure 1). These patients were 60% female and 40% male. The median age of the responders was 61 years. The most prevalent cancers were breast cancer, lung cancer, and colon cancer. Four-fifths of patients started their “patient route” following a visit to a general practitioner or specialist because of symptoms. Half of the patients started their oncological treatment following surgery. In the case of approximately 80% patients, the intention of the treatment was curative (Table 1).

The validation of the questionnaire presents a very good result (kappa: 0.81–1.00; *p* < 0.0001 for seven items, namely, type of “patient route” starting points, gender, age, education, place of residence, professional activity, marital status), a good result (kappa: 0.61–0.80; *p* < 0.0001 for private medical services), and a moderate repeatability result (kappa: 0.41–0.60; *p* < 0.0001 for the date of suspicion). The estimated time distribution was not normal (*p* < 0.001).

The medians of the total interval, the diagnostic interval, and the treatment interval were 10.6 weeks (6.1–17.1 weeks, 25–75% IQR) (IQR: interquartile range), 5.6 weeks (2.7–10.6 weeks, 25–75% IQR), and 5.0 weeks (2.9–7.9 weeks, 25–75% IQR), respectively. The median time of obtaining the results of the histopathological examination was 7 days (4.0–12.0 days, 25–75% IQR). The age was slightly correlated with the total interval (*r* = 0.07; *r*—correlation coefficient), the diagnostic interval (*r* = 0.08), and the treatment interval (*r* = 0.07). The patients who were likely to wait significantly longer (*p* < 0.05) from suspicion to treatment were pensioners (12.0 weeks); those who were diagnosed with head and neck cancer (13.0 weeks), and lung cancer (12.3 weeks); those who started cancer diagnosis from the prevention/screening test (12.0 weeks); those who were treated with radiotherapy or radiochemotherapy (12.7 weeks); those who were treated in center IA (12.4 weeks); and those who did not make use of private medical services (11.6 weeks). The patients who were likely to wait significantly longer (*p* < 0.05) from suspicion to diagnosis were pensioners (6.4 weeks), those who were diagnosed with digestive system cancer (6.6 weeks) and breast cancer (6.4 weeks), and those who were treated in center IIA (7.4 weeks). The patients who were likely to wait significantly longer (*p* < 0.05) from diagnosis to treatment were those who had a primary education (5.3 weeks), those who lived in villages (5.0 weeks) and cities with fewer than 50,000 inhabitants (5.1 weeks), and those who were unemployed (5.8 weeks). The longest waiting time was noted in cases of urinary system cancer (7.4 weeks) (Table 2).

In the multivariate analysis it was noticed that, only in the case of primary tumor localization and the beginning of the method of treatment, was there a significant influence on the total interval. The factors that most affected the diagnostic interval were primary tumor localization, the facilities, and the place of residence. The strongest influences on the treatment interval was estimated, in the case of tumor localization, to be the method of starting treatment and the facilities (Table 3).

**Figure 3:**
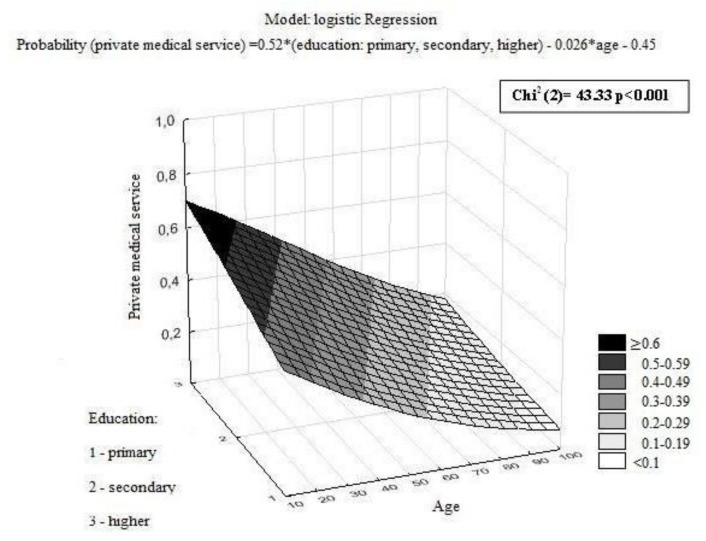
The model of the relation between the probability of using private medical services, age, and education.

Of the patients who were asked about using private medical services during the diagnostic process, 27% admitted to paying for a part of the diagnostic procedures. There was a significant correlation between age and education. Younger patients who had a higher education more-frequently paid for medical services during the diagnostic process (Figure 3). Significant associations were also noted between the use of private medical services and professional activity, marital status, primary tumor localization, and facilities (Table 4).

## 4. Discussion

In Poland, the results of cancer treatment are worse that the European average [4]. Many investigators try to establish the relationship between the delay in waiting time for cancer diagnosis and treatment, and inferior treatment results. This is a controversial subject because of the heterogeneity of published results. Although some analyses confirmed the influence of the delay of oncological therapy on cure and overall survival [7,8,9,10,11,12,13,14]. However, in other analyses, there was no evidence confirming this thesis and the results were often contradictory [15,16,17,18,19,20,21,22,23,24]. A systematic review [13], including over 100,000 patients, confirmed the relationship between the longer waiting time between the initial symptoms and the treatment, reduced survival of breast cancer patients. The extended persistence of symptoms was associated with more advanced diseases. The higher mortality rate of 5–7% was related to the longer waiting time (<3 months vs. 3–6 months). Therefore, the time between the initial symptoms and the treatment should be as short as possible, and should not exceed 3 months [21]. The Netherlands analysis [14] also demonstrated that the longer waiting time between head and neck cancer diagnosis being histopathologically confirmed and the treatment, was significantly related to an increased risk of dying. The three-month delay decreased the overall five-year survival rate by 18%.

This study demonstrated that the waiting time for treatment was too long and, in most cases (60%), exceeded nine weeks (median = 10.6 weeks) from the cancer suspicion. In this study, the histopathological test result was taken as a diagnosis. In this analysis, the waiting time between the suspicion and the diagnosis was 5.6 weeks. Based on the available data, it was difficult to determine precisely how long the cancer diagnostic process lasted. The median waiting time from the diagnosis to the treatment was 5.0 weeks, which is similar to the postulated maximum time in Great Britain. Other authors demonstrated that the median times between the histopathological diagnosis and the treatment for lung, head and neck, bladder, prostate, breast, and colorectal cancer patients, were 4.7, 6.0, 5.3, 8.3, 18.0, and 16.0 weeks, respectively [14,24,25,26,27].

A quarter of the patients in our study had used private medical services during the diagnostic process and they had a significantly shortened waiting time from suspicion to treatment. The patients paid for medical expenses mainly because of general practitioners refusal to give referrals and the extensive waiting time for a specialist visit or diagnostic examination.

This analysis indicated that in cases of younger patients, there was a significantly decreased waiting time from the suspicion to the diagnosis and treatment. In the literature, the influence of age on the waiting time was not clearly confirmed. Stevens et al. [27] estimated that there was a significantly longer time from the suspicion to the treatment of older men with prostate cancer, whereas younger patients with breast and lung cancer waited longer for treatment [24,28]. Van Harten et al. [14] determined that there was a significantly decreased time from diagnosis to treatment of head and neck cancer in a case of younger patients. However, the results of other studies contradicted this, concluding that the younger patients waited significantly longer for treatment [24,25] or that age did not have a significant impact [25,27].

The present analysis did not determine a significant relationship between the sex of the patients and the waiting time. Similarly, Yorio et al. [24] did not find any significant difference in the waiting time from the lung cancer suspicion to the diagnosis and treatment, of patients of different sexes. However, Robertson et al. [28] found there to be a shorter time to the treatment for colorectal cancer in the case of men. In the case of men [14,29] or women [30], depending on the analysis, a significantly increased time between the diagnosis and treatment was determined. However, other studies did not confirm any significant impact of the sex of patients on waiting time [24,25].

In this analysis, the professional activity of the patients significantly impacted the waiting time for the treatment. The longest time from suspicion to diagnosis and treatment was observed in the case of retired patients. Arndt et al. [31] noted that economically active women that were diagnosed with breast cancer waited the shortest period of time between first noticing the symptoms and visiting a doctor. However, the overall differences were insignificant.

This study determined that the only statistically significant difference in the period of time between suspicion and treatment was caused by the type of “patient route” starting points (between the first visit, with symptoms, to a doctor and a preventive or screening test). Robertson et al. [28] suggested that an increase in the symptoms’ severity and a higher frequency of visits to a general practitioner resulted in quicker start to the treatment.

In our study, the primary tumor localization significantly influenced the waiting time from suspicion to diagnosis and treatment, and from diagnosis to treatment. In the multivariate analysis, this factor had the highest impact on every period of time that was analyzed. This could be related to a known variable rate of the natural development of different types of tumors, and consequently, differential diagnostic approaches. In our opinion, the long total interval and diagnostic interval observed in the case of aggressive cancers—such as head and neck, lung, and digestive system cancer—is alarming. The literature confirmed the significant relationship between tumor localization and the time from diagnosis until treatment [29,30]. Wyatt et al. [32] determined that, after breast cancer diagnosis, the prolongation of the waiting time for radiotherapy by one to two months could have significant adverse effects on treatment outcomes. In the case of prostate cancer, however, even a delay of several months did not negatively influence the local control.

This analysis indicated that the significant differences of the waiting time from suspicion to diagnosis and treatment, was dependent on the facilities. The published analysis reported that there was a shorter waiting time in private centers than in public centers [24,28].

Some of the analyses demonstrated that there was no difference in waiting time between the subgroups of patients living in cities and villages [33,34]. The patients from bigger cities waited significantly shorter times from suspicion to diagnosis than the patients living in smaller cities. In our analysis, the waiting time from suspicion to treatment did not differ significantly depending on the place of residence. However, a significant difference was noted in the waiting time from diagnosis to treatment (the longest was in the case of inhabitants of villages and small cities).

## 5. Conclusions

In conclusion, the waiting time for the treatment of cancer patients in Poland is too long—exceeding nine weeks in 60% of the cases. A quarter of the patients used private medical services (which is paid for by the patients and not reimbursed) during the diagnostic process, in order to reduce the diagnosis time. The patient-related factors were not the main cause of the prolongation of the waiting time between diagnosis and treatment. The most significant influences were determined to be the tumor type and factors related to the system. Decreasing the waiting time for oncological therapy could be one of the strategic goals leading to the improvement of the relative survival rate of cancer patients in Poland. It is challenging to find a solution to decrease the time from the suspicion to the cancer diagnosis. It is likely that reasonable administrative regulations with some maximum time intervals, as is the case in some European countries, such as Great Britain, Denmark, and the Czech Republic, would be effective. As is the case in the UK, it is vital to remove limits on the reimbursement of oncological services. Nowadays, the reimbursement in Poland is limited or postponed in both the diagnostic and therapeutic fields of oncology. In general, the Polish public health care system is underfinanced in comparison with other European countries, including countries from the former Eastern Bloc. Additionally, the coordination of the diagnostic and therapeutic processes should be improved on various levels of the healthcare system. Finally, Poland has too few physicians per general population when compared with EU standards. Consequently, Polish physicians and other health care professionals are heavily overloaded with bureaucracy and paper work.

The analysis of the waiting times for oncological treatment is difficult because of the subjective assessment of the date that the first symptoms appeared by patients, and the inaccuracy of the retrospective analysis of patients’ medical documentation. To our knowledge, this study was the first of its kind conducted in Poland thus far. Despite of some methodological shortcomings listed above, these results appear to be reliable and could compared to similar analyses conducted in the future.

## Figures and Tables

**Table 1:** Characteristics of the patients included in the analysis.

Characteristic	Patients Included in Total Interval Analysis		Patients Included in Diagnostic and Treatment Interval Analysis	
	*n*	(%)	*n*	(%)
All patients	1143	(100)	789	(100)
Age (years)	median 61; range 21–91		median 60; range 21–91	
Gender				
Female	683	(60)	477	(60)
Male	460	(40)	312	(40)
Education				
Primary	256	(22)	179	(23)
Secondary	729	(64)	503	(64)
Higher	158	(14)	107	(13)
Place of residence				
City >500,000	65	(6)	43	(5)
City 101,000–500,000	282	(25)	208	(26)
City 50,000–100,000	118	(10)	78	(10)
City <500,000	342	(30)	235	(30)
Village	336	(29)	225	(29)
Professional activity				
Student	2	(0.2)	2	(0.3)
Active	371	(33)	268	(34)
Unemployed	82	(7)	58	(7)
Pensioner	688	(60)	461	(59)
Marital status				
Married	781	(68)	536	(68)
Single	166	(15)	117	(15)
Widow/er	196	(17)	136	(17)
Primary tumor localization				
Breast	317	(28)	240	(30)
Lung	224	(20)	179	(23)
Colon	164	(14)	95	(12)
Head and Neck	123	(11)	98	(13)
Female reproductive organs	119	(10)	73	(9)
Digestive system (upper section)	60	(5)	41	(5)
Urinary system (without prostate)	40	(4)	20	(3)
Brain	35	(3)	9	(1)
Others	61	(5)	34	(4)
Type of “patient route” starting points				
Symptoms	917	(80)	655	(83)
Privention/screening	159	(14)	113	(14)
Follow-up	67	(6)	21	(3)
Method of treatment beginning				
Surgery	583	(51)	304	(39)
Radiotherapy/radiochemotherapy	316	(28)	277	(35)
Chemotherapy	241	(21)	205	(26)
Hormonal therapy	3	(0.3)	3	(0.4)
Treatment intention				
Curative	899	(79)	650	(82)
Palliative	240	(21)	138	(18)
No data	4	(0.3)	1	(0.1)
Facilities				
IA	368	(32)	270	(34)
IB	223	(19)	143	(18)
IC	147	(13)	89	(11)
IIA	155	(14)	88	(11)
IIIA	134	(12)	93	(12)
IIIB	116	(10)	106	(14)
Private medical services				
Yes	248	(22)	173	(22)
No	683	(60)	477	(60)
No data	212	(18)	139	(18)

**Table 2:** The statistics (median, IQR) of different periods of time and the differences between subgroups of patients.

Variable Name	Total Interval (Weeks)			Diagnostic Interval (Weeks)			Treatment Interval (Weeks)		
	Median	(25–75% IQR)	*p*-Value	Median	(25–75% IQR)	*p*-Value	Median	(25–75% IQR)	*p*-Value
All patients	10.6	(6.1–17.1)		5.6	(2.7–10.6)		5.0	(2.9–7.9)	
Gender			0.867			0.215			0.078
Female	10.6	(6.1–17.0)		5.4	(2.4–10.4)		4.9	(2.9–7.4)	
Male	10.6	(6.0–17.3)		6.0	(3.0–11.1)		5.1	(3.0–8.4)	
Education			0.076			0.383			0.019
Primary (A)	12.2	(6.7–17.1)		5.3	(3.1–10.1)		5.3	(3.1–9.4)	
Secondary (B)	10.4	(6.1–17.1)		5.7	(2.7–10.7)		5.0	(2.9–7.7)	
Higher (C)	9.2	(5.4–16.0)		5.3	(2.0–11.1)		4.1	(2.9–6.4)	
							(*p* < 0.05 post hoc between A/C)		
Place of residence			0.061			0.066			0.016
City >500,000 (A)	8.6	(5.7–16.9)		3.4	(2.1–6.6)		4.4	(3.0–8.1)	
City 101,000–500,000 (B)	10.6	(5.7–17.1)		5.3	(2.4–10.5)		5.1	(2.9–8.6)	
City 50,000–100,0000 (C)	8.6	(5.4–17.6)		6.4	(2.6–11.0)		3.6	(2.0–6.4)	
City <50,000 (D)	11.7	(7.0–17.9)		6.4	(2.6–11.3)		5.1	(3.0–7.7)	
Village (E)	10.8	(6.2–16.9)		5.7	(3.3–11.0)		5.0	(3.0–7.6)	
							(*p* < 0.05 post hoc between B/C, C/D, C/E)		
Professional activity			0.006			<0.001			0.041
Student *	7.6	(6.6–8.7)		3.4	(0.6–6.1)		4.3	(2.6–6.0)	
Active (A)	9.3	(5.7–16.3)		4.6	(2.1–9.7)		4.6	(2.9–7.1)	
Unemployed (B)	9.4	(6.1–14.6)		4.4	(2.0–8.6)		5.8	(3.0–8.3)	
Pensioner (C)	12.0	(6.4–17.7)		6.4	(3.3–11.4)		5.1	(2.9–8.1)	
	(*p* < 0.05 post hoc between A/C)			(*p* < 0.05 post hoc between A/C)					
Marital status			0.189			0.514			0.994
Married	10.9	(6.0–17.3)		5.7	(2.7–11.2)		5.0	(2.9–7.6)	
Single	9.7	(5.3–16.0)		5.1	(2.3–9.6)		4.7	(2.6–8.3)	
Widow/er	10.6	(6.9–17.5)		5.9	(3.0–9.3)		4.6	(3.0–7.9)	
Primary tumor localization			<0.001			<0.001			<0.001
Breast (A)	10.9	(6.3–16.0)		6.4	(3.3–10.5)		4.1	(2.6–6.3)	
Lung (B)	12.3	(7.7–19.0)		6.3	(3.3–9.7)		5.0	(2.4–8.7)	
Colon (C)	9.1	(5.2–16.7)		5.3	(2.4–14.0)		4.4	(2.6–6.3)	
Head and Neck (D)	13.0	(6.7–20.1)		5.5	(3.0–12.3)		6.1	(3.7–9.3)	
Female reproductive organs (E)	8.6	(5.3–12.9)		2.0	(1.3–4.0)		5.9	(3.9–8.4)	
Digestive system (upper section) (F)	11.0	(5.3–17.4)		6.6	(3.3–10.4)		5.1	(3.1–8.6)	
Urinary system (without prostate) (G)	9.7	(5.2–21.6)		4.9	(2.9–9.5)		7.4	(6.0–11.9)	
Brain (H)	6.1	(1.9–12.0)		5.9	(2.4–13.0)		4.9	(2.4–5.7)	
Others (I)	14.6	(6.9–20.6)		6.2	(3.7–16.0)		6.2	(3.3–9.6)	
	(*p* < 0.05 post hoc between B/C, B/E, B/H, D/E, D/H, E/I, H/I)			(*p* < 0.05 post hoc between B/E, C/E, D/E, E/G, E/F, A/E, E/I)			(*p* < 0.05 post hoc between B/G, C/D, C/G, A/D, A/E, A/G)		
Type of “patient route” starting points			0.030			0.083			0.886
Symptoms (A)	10.1	(6.0–17.0)		5.3	(2.6–10.4)		5.0	(2.9–8.0)	
Prevention/screening (B)	12.0	(7.9–18.9)		6.9	(3.7–11.0)		4.9	(3.3–7.1)	
Follow-up (C)	10.1	(4.6–18.7)		4.3	(1.9–13.0)		4.1	(2.9–7.0)	
	(*p* < 0.05 post hoc between A/B)								
Method of treatment beginning			<0.001			0.335			<0.001
Surgery (A)	8.9	(4.9–15.9)		5.9	(2.8–11.4)		4.1	(2.7–6.3)	
Radiotherapy/ radiochemotherapy (B)	12.7	(8.0–19.6)		5.0	(2.6–10.1)		6.3	(4.0–9.6)	
Chemotherapy (C)	12.1	(7.1–17.0)		6.1	(2.6–10.0)		4.7	(2.4–7.4)	
Hormonal therapy *	14.1	(5.3–17.0)		7.9	(1.4–10.1)		4.0	(3.9–9.1)	
	(*p* < 0.05 post hoc between A/B)						(*p* < 0.05 post hoc between A/B, B/C)		
Treatment intention			0.598			0.148			0.524
Curative	10.6	(6.3–17.0)		5.6	(2.7–10.3)		4.9	(2.9–7.9)	
Palliative	10.5	(5.2–18.1)		6.5	(3.0–12.4)		5.1	(2.4–7.4)	
Facilities			<0.001			<0.001			0.073
IA (A)	12.4	(7.6–16.9)		6.3	(3.0–11.6)		5.1	(2.9–7.9)	
IB (B)	9.3	(5.7–17.3)		5.3	(2.1–10.0)		5.0	(3.1–8.3)	
IC (C)	9.4	(4.9–17.7)		6.4	(3.1–12.6)		5.0	(3.0–7.4)	
IIA (D)	10.7	(5.7–19.0)		7.4	(3.6–12.6)		3.6	(2.4–6.3)	
IIIA (E)	8.6	(4.9–13.0)		3.3	(2.1–5.6)		4.1	(2.4–7.9)	
IIIB (F)	12.1	(7.9–19.9)		6.2	(3.4–11.4)		5.5	(3.3–8.4)	
	(*p* < 0.05 post hoc between A/E, E/F)			(*p* < 0.05 post hoc between A/E, B/E, C/E, D/E, E/F)					
Private medical services			0.011			0.133			0.284
Yes	9.4	(5.1–17.1)		5.0	(2.3–11.1)		5.1	(2.9–7.4)	
No	11.6	(6.9–17.7)		6.1	(3.0–11.1)		5.1	(3.0–8.1)	

IQR—interquartile range; *p*—probability of Mann–Whitney or Kruskal–Wallis test; post hoc—post hoc Dunn’s test; * Student and hormonal therapy subgroups were excluded from the analysis.

**Table 3:** The association between the predicted variables and the waiting time using the generalized linear model (GRM).

Time	Variables	*p*-Value	Akaike Information Criterion (AIC)	Log-Max. Likelihood	*p*-Value
Total interval	Primary tumor localization	<0.001	6515.93	61.53	<0.001
	Method of treatment beginning *	0.003			
Diagnostic interval	Primary tumor localization	<0.001	3959.99	129.51	<0.001
	Facilities	<0.001			
	Place of residence	0.019			
Treatment interval	Primary tumor localization	<0.001	3416.75	122.48	<0.001
	Method of treatment beginning *	<0.001			
	Facilities	<0.001			

* Hormonal therapy subgroup was excluded from the analysis.

**Table 4:** The association between the predicted variables and using private medical services.

Variable Name	Private Medical Service		Public Medical Service		*p*-Value
	*n*	(%)	*n*	(%)	
All patients	248	(27)	683	(73)	
Gender					0.051
Female	155	(29)	378	(71)	
Male	93	(23)	305	(77)	
Education					<0.001
Primary	34	(16)	174	(84)	
Secondary	160	(27)	433	(73)	
Higher	54	(42)	76	(58)	
Place of residence					0.106
City >500,000	10	(22)	35	(78)	
City 101,000–500,000	67	(32)	141	(68)	
City 50,000–100,000	26	(29)	65	(71)	
City <50,000	67	(22)	239	(78)	
Village	78	(28)	203	(72)	
Professional activity					<0.001
Student *	1	(50)	1	(50)	
Active	125	(39)	192	(61)	
Unemployed	11	(17)	52	(83)	
Pensioner	111	(20)	438	(80)	
Marital status					0.007
Married	185	(29)	463	(71)	
Single	37	(29)	89	(71)	
Widow/er	26	(17)	131	(83)	
Primary tumor localization					<0.001
Breast	73	(32)	158	(68)	
Lung	24	(13)	156	(87)	
Colon	43	(32)	93	(68)	
Head and Neck	20	(18)	90	(82)	
Female reproductive organs	29	(31)	66	(69)	
Digestive system (upper section)	18	(32)	38	(68)	
Urinary system (without prostate)	18	(49)	19	(51)	
Brain	11	(33)	22	(67)	
Others	12	(23)	41	(77)	
Treatment intention					0.113
Curative	199	(28)	511	(72)	
Palliative	49	(23)	168	(77)	
Facilities					<0.001
IA	77	(21)	291	(79)	
IB	57	(40)	84	(60)	
IC	52	(35)	95	(65)	
IIA	22	(29)	55	(71)	
IIIA	20	(24)	62	(76)	
IIIB	20	(17)	96	(83)	

*p*—probability of chi-square test. * Student subgroup was excluded from the analysis.
